# Utilization of electronic health records for the assessment of adiponectin receptor autoantibodies during the progression of cardio-metabolic comorbidities

**DOI:** 10.46439/autoimmune.1.004

**Published:** 2020

**Authors:** Michael J. Pugia, Meeta Pradhan, Rong Qi, Doreen L. Eastes, Anna Vorsilak, Bradley J. Mills, Zane Baird, Aruna Wijeratne, Scott M. McAhren, Amber Mosley, Anantha Shekhar, Daniel H. Robertson

**Affiliations:** 1 Bioanalytical Research Core, Indiana Biosciences Research Institute, Indianapolis IN, USA; 2 Applied Data Sciences Center, Indiana Biosciences Research Institute, Indianapolis IN, USA; 3 Indiana University School of Medicine, Indianapolis IN, USA; 4 Eli Lilly and Company, Lilly Corporate Center, Indianapolis IN, USA

## Abstract

**Background:**

Diabetes is a complex, multi-symptomatic disease whose complications drives increases in healthcare costs as the diabetes prevalence grows rapidly world-wide. Real-world electronic health records (EHRs) coupled with patient biospecimens, biological understanding, and technologies can characterize emerging diagnostic autoimmune markers resulting from proteomic discoveries.

**Methods:**

Circulating autoantibodies for C-terminal fragments of adiponectin receptor 1 (IgG-CTF) were measured by immunoassay to establish the reference range using midpoint samples from 1862 participants in a 20-year observational study of type 2 diabetes and cardiovascular arterial disease (CVAD) conducted by the Fairbanks Institute. The White Blood Cell elastase activity in these patients was assessed using immunoassays for Bikunin and Uristatin. Participants were assigned to four cohorts (healthy, T2D, CV, CV+T2D) based on analysis of their EHRs and the diagnostic biomarkers values and patient status were assessed ten-years post-sample.

**Results:**

The IgG-CTF reference range was determined to be 75–821 ng/mL and IgG-CTF out-of-range values did not predict cohort or comorbidity as determined from the EHRs at 10 years after sample collection nor did IgG-CTF demonstrate a significant risk for comorbidity or death. Many patients at sample collection time had other conditions (hypertension, hyperlipidemia, or other risk factors) of which only hypertension, Uristatin and Bikunin values correlated with increased risk of developing additional comorbidities (odds ratio 2.58–13.11, P<0.05).

**Conclusions:**

This study confirms that retrospective analysis of biorepositories coupled with EHRs can establish reference ranges for novel autoimmune diagnostic markers and provide insights into prediction of specific health outcomes and correlations to other markers.

## Background

Diabetes is an independent risk factor for cardiovascular arterial disease (CVAD), kidney disease, liver disease, Alzheimer’s disease, and many other comorbidities, of which the costs are increasing at >25% per year, with 380 million people likely to be affected by 2025 [1]. CVAD has the greatest economic burden, affecting one in four American adults and accounting for 6 million hospitalizations per year as well as nearly 40% of all deaths (~17 million per year). Patients with both diabetes and CVAD exhibit significantly higher hazard ratios for additional complications than those with diabetes alone [2,3].

Insulin resistance and chronic inflammation are strongly associated with the progression of metabolic syndrome and CVAD in diabetes patients [4,5]. These are complex, multi-factorial conditions and numerous biomarkers have been proposed, but few have proven effective for patient management [5]. An ideal biomarker would provide risk assessment across all individuals and accurately predict progression to various complications. However, current methods are non-specific and cannot predict progression without complex rule-in and rule-out algorithms. Although antihyperglycemic medications and standards of care for the management of weight, diet, glycosylated hemoglobin, micro-albuminuria, and albuminuria significantly reduce the risk of CVAD, there remains an urgent need to improve the health economics of diabetes [6–8].

Clinical outcome studies of diabetic complications can be long and expensive, with the death rate at 1–3% per year [1,9]. The cost of diagnostic marker assessment is prohibitive for relative risk analysis unless retrospective studies are used to confirm progression outcomes. Additionally, investigational assessments based on cohort comparison often introduce selection bias and cannot justify the verification and validation costs for prospective analysis [10]. Retrospective analysis also lacks real-world content because it predefines the outcome and cannot adjust for wider results. The use of electronic heath records (EHRs) provides real-world data and evidence, offering a promising and more economical method for the assessment of comorbidities, as recently shown for the prediction of chronic kidney disease [11].

Diagnostic accuracy can be improved by the detection of autoantibodies, as shown for autoantibodies against cytokines that predict autoimmune disease and tissue injury caused by autoreactive antibodies and T cells [12,13]. Autoantibodies can be quantified using anti-antigen antibodies, as shown for the adiponectin receptor C-terminal fragment (AdipoR1 CTF_344–375_) antigen, which circulates freely in the plasma of healthy individuals but not in some diabetes patients (P>0.001) [14]. Proteomic analysis of autoantigens is complex and difficult to translate due to the low transient concentrations (<5 ng/mL) and the need to use both stable isotope standards and monoclonal antibodies for capture [15–17]. However, once characterized, autoantigens can lead to the identification of new receptor domains, such as the highly conserved AdipoR1 CTF_351–362_ fragment, a strong non-competitive inhibitor of insulin-degrading enzyme (IDE) [18]. Autoantibodies can be measured routinely in human samples due to their high non-transient concentrations, including autoantibodies against AdipoR1 CTF_344–375_ (IgG-CTF) with a concentration range of 5–4900 ng/mL [18].

Measuring the diagnostic significance of autoantibodies as a personalized response to disease is difficult given the long time needed for the disease to develop. However, the interaction of AdipoR1 CTF_351–362_ with IDE has diagnostic potential because mechanistic and drug studies have confirmed an impact of IDE on the insulin response in type 2 diabetes and CVAD [19–22]. AdipoR1, a G-protein coupled receptor, enhances glucose uptake and fatty acid oxidation in muscle, suppresses glucose output by the liver, and increases insulin sensitivity [23–26]. Low adiponectin levels predict a higher risk of type 2 diabetes and CVAD [27–35].

Autoantibodies against AdipoR1 CTF_344–375_ have been recently shown to develop during the onset of type 1 diabetes in the non-obese diabetic mouse model and correlated with loss of AdipoR1 signaling of AMBK in the pancreas [36]. Affected mice exhibited increased AdipoR1 shedding with marked increase of White Blood Cells in the pancreas and increased proteolytic activity, especially neutrophilic elastase [36]. The direct impact increased proteolytic activity on AdipoR1 auto antibody formation remain un-resolved. The quantification of Bikunin and Uristatin by immunoassay is a convenient means to measure any increased human neutrophilic elastase in human patients due to inflammation, infection, cardiovascular disease, and kidney diseases [37–40]. Therefore, the measurements of Bikunin and Uristatin offered an assessment of increased proteolytic activity when testing for diagnostic significance of AdipoR1 CTF_344–375_ autoantibodies.

The Fairbanks Institute for Healthy Communities established a biorepository comprising samples from more than 1900 Indianapolis-area type 2 diabetes and CVAD patients and controls over a 2-year period. Today, this sample bank combined with the patient’s EHRs allows the analysis of novel biomarkers and their utility in predicting future diabetic complications. We assessed the levels of IgG-CTF autoantibodies, Bikunin and Uristatin in these samples of healthy controls, diabetic, and CVAD patients using the Fairbanks biorepository. These samples, in combination with real-world outcomes based on EHR data covering the subsequent 10 years post sample-collection, established IgG-CTF reference ranges and understanding of the diagnostic significance of autoantibodies for the risk of additional comorbidities.

## Methods

### Fairbanks Institute biorepository

The Fairbanks Institute biorepository (NCT01386801, NCT00741416) was created as an extensively annotated sample repository for hypothesis-driven research that would lead to advances in the diagnosis, treatment and prevention of diseases affecting the population of Indiana. The study was conducted in accordance with Indiana University’s Internal Review Board (Protocol 1011003179: Multicenter Research Study to Build a Repository that will allow Researchers to Study Chronic Diseases in the Population of Central Indiana). All participants were 18 years or older and gave consent to provide samples.

The participants of this study were originally recruited to the type 2 diabetes cohort, CVAD cohort or healthy controls to these cohorts as defined by the criteria listed below during timeframe of sample collection (2007–2010). Study subjects in the diabetes cohort were recruited based on an EHR-confirmed history of at least one of the following: fasting blood glucose ≥ 126 mg/dL on two separate occasions; random (non-fasting) blood glucose ≥ 200 mg/dL on two separate occasions; blood glucose >200 mg/dL at 2 h during a standard oral glucose tolerance test; or hemoglobin A1c (HbA1c) ≥ 6.5%. Study subjects in the CVAD cohort were recruited based on an EHR-confirmed history of at least one of the following: angioplasty, with or without stent placement; coronary artery bypass graft surgery; diagnostic angiogram; or positive catheterization results showing ≥ 50% occlusion. Healthy controls for the study were recruited based on having no confirmed history of any form of diabetes (as defined above) and not having a history of CVAD or other risk factors.

Biological specimens were collected from 1966 individuals (n=724 CVAD, n=590 diabetes, n=652 controls) as follows: three 10-mL EDTA tubes for plasma, 14 mL of urine, three 10-mL serum separation tubes (red tops) for serum, and two 3-mL PAXgene tubes for RNA. Specimens were divided into 0.5-mL aliquots and were stored at −80 °C (BioStorage Inc, Indianapolis, IN). Urine specimens were only collected from the diabetes group and half of the control subjects. Of these specimens, 1862 individuals had a complete set of samples with corresponding EHRs that could be used for biomarker assessment in this study. Table 1 details the patient demographics at the time of sample collection.

### Collection of EHRs

When the Fairbanks Institute was launched, the Regenstrief Institute, Inc. (Indianapolis, IN) was responsible for providing access to the associated EHRs of the patients for research use and maintain the linkage of sample IDs to patient IDs within the Indiana Network of Patient Care (INPC) EHR repository. The Indiana Health Information Exchange is the organization responsible for collecting the EHRs into the INPC database from the participating healthcare organizations within Indiana. For the available samples, all data over 20 years was requested, including demographics, diagnosis codes, medications, clinical laboratory results, and procedures (for n=1907 patients). No *a priori* filtering of the health data was requested. The data were securely delivered to the IBRI as a set of de-identified data extracts according to Health Insurance Portability and Accountability Act safe harbor rules.

### Initial analysis of EHR data

EHRs were cleaned and analyzed to understand the patient disease state at sample collection by mapping diagnosis codes (ICD-9/ICD-10) to diseases. Any recorded diagnosis code prior to or within 30 days after sample collection attributed that disease to that individual at the time of sample collection. The patients and control diagnosis codes were reassessed 5 years after sample collection (Table 1). The number of medications was computed by mapping the National Drug Code to pharmaceutical class using the National Library of Medicine RxNorm [41]. Due to the variability in duration of prescriptions, a prescription was counted if filled 180 days before or after sample collection. Finally, the clinical variables from the EHRs were normalized relative to names and units of measures, outliers removed, and values computed for each patient as the mean of values within 180 days of the sample collection date.

Diagnostic testing performed during patient care after sample collection was obtained from the EHRs and matched with the demographics for (n=1847) patients and controls who remained in the system (Table 2). Because real-world can be incomplete, not all data was available for each diagnostic test or individual. The absence of data does not necessarily mean the absence of the condition, so the data in Table 2 are considered minimum values. Clinical parameters with fewer than 10 patients per group were discarded due to lack of data. Means and standard deviations were used from the total sum of results for each patient and across the available records.

### Statistical Analysis of EHRs

The affected groups were compared to the control groups for all EHR diagnostic measurements (n=1847) (Table 2). For this study, based on EHR analysis, the patients were assigned to one of four cohorts at the time of sample collection, regardless of their original cohort assigned from the Fairbanks study designation: Control Group, Type 2 Diabetes (T2D), Cardiovascular (CV), and Type 2 Diabetes and Cardiovascular (CV+T2D). The p value for differences between the affected and control groups was calculated using the normal approximation to the binomial distribution with continuity correction. For quantitative factors (e.g. age and diagnostic tests), the count, mean, standard deviation, minimum and maximum values, and calculated p values were determined for differences between group means assuming a *t*-distribution. Before calculating the *t*-value, the group standard deviations were compared using the F distribution. The clinical variables associated with the cohorts defined in Table 1 were analyzed to understand their significance within and across the cohorts. To understand the significance of the variable with respect to each cohort, between cohorts and across cohorts, the data were processed by univariate and bivariate analysis and ANOVA. Each variable was analyzed for significance across cohorts using an independent samples *t*-test. Table 2 shows the significant p values for each variable. Differences between the affected and control groups were deemed statistically significant if the corresponding p values were less than 0.001 (99.9%), 0.01 (99%) or 0.05 (95%).

### Analysis of EHR outcomes

**Figure 1:**
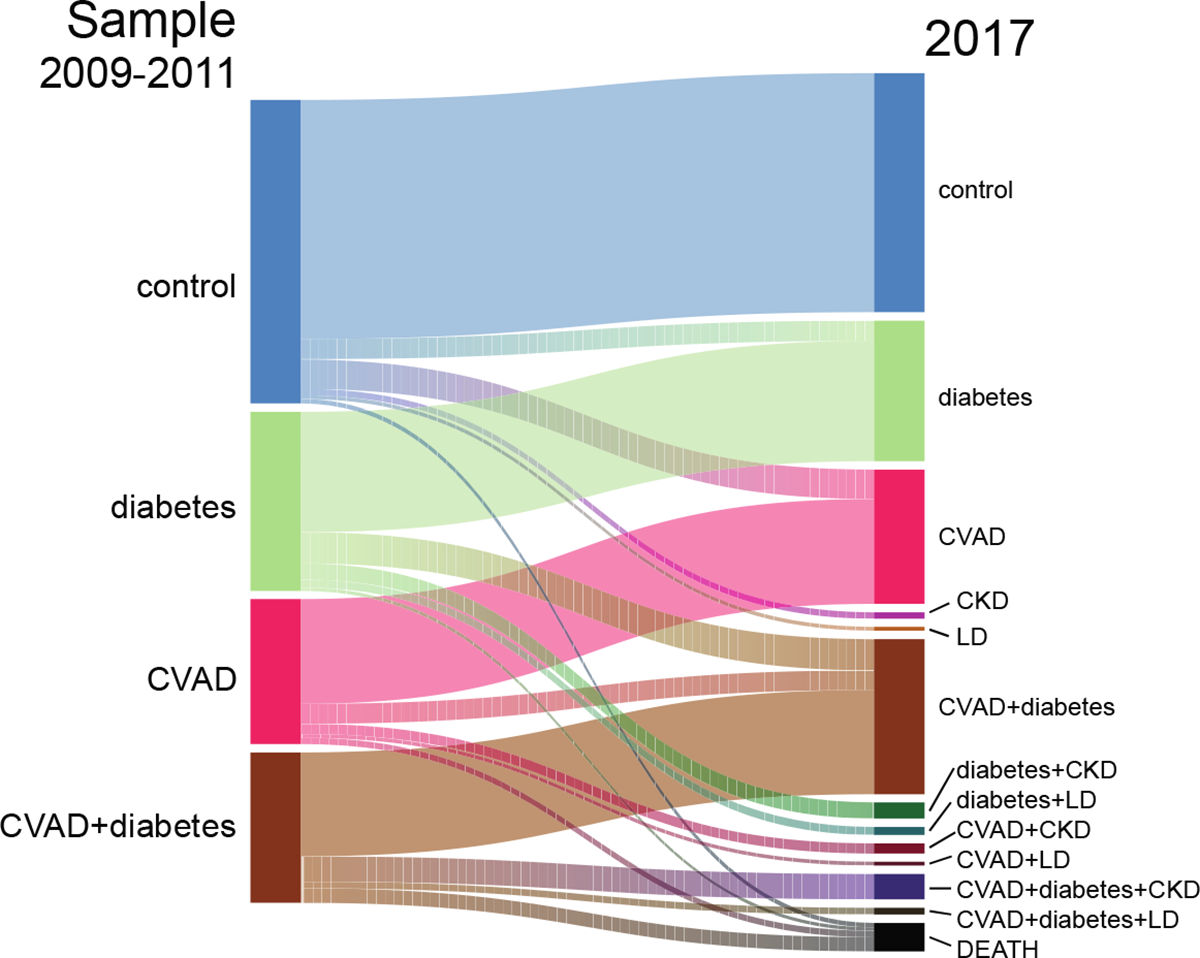
Sankey flow diagram showing the transition from initial disease state at sample collection to final outcome based on EHR data for the 1847 patients. The bars represent the number of individuals within that disease state and the width of the connections represents that number of the individuals transitioning to the new final state after sample collection. The number of individuals with hypertension alone, hyperlipidemia alone, or both, was also computed for these cohorts at sample collection but the data are not shown. These numbers are for the control (29,78,39), diabetes-T2D (28, 82, 182), CVAD-CV (16, 77, 242) and CV+diabetes/CV+T2D (14, 37, 315) cohorts, respectively.

The EHRs of total patients (n=1847) and cohorts were assessed for disease indications and the presence of hypertension (HT) and/or hyperlipidemia (HLD) prior to sample collection. The subsequent development of comorbidities was assessed by EHR data analysis to confirm the development of type 2 diabetes (T2D) (n=174), cardiovascular (CV) (n=140), chronic kidney disease (CKD, n=136), or liver disease (LD, n=53). The development of multiple comorbidities such as CV+T2Ds (n=39), T2D+CKD (n=32), T2D+LD (n=10), and CV+CKD (n=29) was also recorded. The disease progression from initial sample collection to final outcome based on available EHR data is depicted in the Sankey flow diagram (Figure 1). Additional phenotypes combining more than two comorbidities represented less than 0.7% of the patients and their significance could not be assessed.

### New marker measurements

Available plasma specimens (n=1783) were measured for IgG-CTF using a previously described sandwich ELISA based on a monoclonal antibody specific for AdipoR1 CTF (ATCC 444–1D12.1H7) and a human IgG-specific antibody conjugated to ALP [14] (Table 3). Plasma (~0.5 mL) was thawed to room temperature, and 10 μL was diluted with 480 μL of stabilization buffer (PBS supplemented with 1% BSA, 0.1 M citrate and 0.01 M EDTA, pH 6.4). Diluted specimens were tested as above or stored at −80 °C.

Proteomics analysis confirming the autoantigen presence in the samples was performed using an anti-AdipoR1 CTF antibody (444 clone, 4.3 mg/mL) directly conjugated to biotin to extract the IgG-CTF from plasma samples, and the free CTF was then released using Tris base (pH ≥8.3) as previously described [14,18]. The free CTF was directly measured against synthetic AdipoR1 CTF_345–375_ (31-mer peptide) calibrator taken to represent the elastase cleavage site and isotope label internal standard (Celtek Biosciences, Franklin, TN) to quantify down to limit of quantitation of 50 fmoles (Proteomic Core Procedure IU School of Medicine). Additionally, western blot analysis of plasma samples confirmed IgG-CTF as the key bound forms by the presence of gamma heavy chains (50–55 kDa) and kappa light chains (26–28 kDa) but a lack significant direct attachment to other proteins as previously reported [14].

The presence of any infection or inflammation as indicated by elastase release of Bikunin and Uristatin (Table 3) was determined for all available urine samples (n=663) collected only from the T2D cohort and all available plasma samples (n=1713) by enzyme-linked immunosorbent assay (ELISA) as previously described [37]. Samples were thawed to room temperature, 10 μL was transferred to duplicate lanes of a polypropylene sample plate, diluted and assayed as previously described [37]. The plate was stored at 4 °C for testing within 24 h or at −80 °C for future testing (up to 5 years).

### Comorbidity statistical analysis

Odds ratios were calculated to compare patients with new disease from those with unchanged clinical profiles when abnormal biomarkers, HT or HLD were present. The morbidity for all cases was determined (n=68) and used to calculate survival odds ratios. Patients developing T2D, CV, CKD or LD comorbidities at any time after sample collection were considered as the combined group of patients who progressed to additional comorbidities. Patient deaths were assessed across all four patient groups for sample sizes above n=10. The significance of odds ratios was estimated using standard error for the odds ratios was based on the sample size.

## Results

### Participant characteristics

Patients were equally represented in terms of gender (~50%) and initial disease groupings (~20% T2D, CV and CV+T2D) (Table 1). Age (30s to 80s) and race (80% white) were also consistent across the groups. Medications were consistent with expected standards of care. The proportion of patients with T2D, CV and CV+T2D increased significantly in the 5 years post sample collection (Table 1).

### Laboratory Measurements

The types of diagnostic tests used in this study agreed with the standards of care that would be expected for patients with T2D and CV over this study period. Most patients had multiple tests for key monitors such as glucose, complete blood cell count, lipid panels, and kidney function. The additional measurements indicated the correlation between parameters and conditions are shown in Table 2. Fasting blood glucose levels and HbA1c were elevated for T2D but not CV patients. The spot glucose ranges were significantly elevated for T2D and only slightly elevated for CV patients. The kidney function results based on estimated glomerular filtration rate (eGFR) values were significantly worse for CV patients but not T2D patients. Only the CV and diabetes groups had higher mean blood urea nitrogen (BUN) values, lower serum creatine, and lower plasma albumin as expected given the worse renal conditions. Micro-albuminuria testing (albumin/creatine ratio) was measured too rarely to be significantly assessed in the EHR data.

The mean neutrophil counts were significantly elevated in both T2D and CV patients. Monocyte counts were lower in T2D and higher in CV patients. Total lipids, LDL, and HDL were significantly lower in T2D and CV patients compared to controls (patients were generally on lipid-lowering medications). Blood pressure ranges showed no significant differences. Triglyceride levels were highest in T2D patients followed by CV patients and were consistently higher than the control group.

### New biomarker testing

The observed reference range of IgG-CTF was 75–821 ng/mL (mean 237 ng/mL, standard deviation 156) with 2.5% of participants exhibiting elevated autoantibodies (821–7892 ng/mL) and 11% lacking autoantibodies (<75 ng/mL). No significant differences were observed between control group, T2D, CV, and CV+T2D cohorts when autoantibodies were absent (all groups, 2.9–5.1%) or present at elevated levels (all groups, 0.4–0.9%) (Table 3).

Uristatin immunoassay urine values ranged from 0.1 to 278.9 mg/L, with 84% of the patients within the normal range <7.5 mg/L at sample collection (Table 3–B). The 98% reference range of <7.5 mg/L for normal results was previously determined for 6292 patients lacking infection, inflammation or kidney disease (37–40). In 19% samples (n=127) the level of Uristatin was ≥7.5 mg/L. The mean values did not differ significantly between the control, T2D, and CV+T2D cohorts. Elevated Uristatin values (≥7.5 mg/L) were observed in all groups.

Bikunin immunoassay plasma values ranged from 0.0 to 2.43 μg/mL. Previously we reported 94% of the CV patients and 97 % of metabolic syndrome patients have Bikunin values in the normal range of <1.4 μg/mL (n= 188 patients) [40]. In agreement, we found the 93% of the 1932 patients here had values of <1.4 μg/mL (Table 3–C). The mean values did not differ significantly between patient groups and elevated Bikunin ≥1.4 μg/mL (7%, n=119 patients) was observed in all groups (control group, CV and T2D).

### Diagnostic phenotyping and impact

The highest mortality rates (9.5% over 10 years) were observed for T2D patients who progressed to CV followed by CV-only patients (4.4%) while the mortality rates for T2D patients who developed no comorbidities (1.9%) and control group (1.5%) were significantly lower over the same 10-year period (Table 4). For the control group cohort, the most likely new comorbidities were CV (9.7%), T2D (6.7%), CKD (2.1%) and LD (1.2%). For the CV cohort, the most likely new comorbidities were diabetes (13.7%), CKD (7.0%) and LD (2.6%). For the T2D cohort, the most likely new comorbidities were CV (17.4%), CKD (8.9%) and LD (4.5%). For the CV+T2D cohort, the most likely new comorbidities were CKD (16.8%) and LD (4.5%).

Using only the statistically significant results from Table 4, patients with HT in the Control Group and CV cohorts were found to be more likely to develop new comorbidities (odds ratio 4.36–5.34). Additionally, T2D patients positive for HLD appear to have delayed progression to comorbidities, but overall any individual positive with HLD showed increased likelihood of death. Elevated uristatin (≥7.5 mg/L) showed no significant likelihood to progress to new comorbidities across any cohorts (Table 4–A). Patients lacking IgG-CTF (≤75ng/mL) were no more likely to progress to comorbidities (odds ratio 0.73–1.48) than those with elevated IgG-CTF (≥821ng/mL) (odds ratio 0.87 to 1.93) (Table 4–A). However, there were no observations within this study of anyone with elevated IgG-CTF progressing to death and lowered IgG-CTF showed increased odds ratio for risk of death, but the p value as only at 93% confidence interval. In this study, due to lack of observations we cannot place statistical significance on either of these odds ratios and a larger study would be needed to confirm this protective nature of elevated IgG-CTF or the increased risk to death from lowered Ig-CTF (Table 4–A). T2D patients with elevated Uristatin (>7.5 mg/L) and CV patients with elevated Bikunin (>1.4 μg/mL) were both more likely to progress to comorbidities with significant odds ratios of 13.11 and 4.44, respectively (Table 4–B).

## Conclusions

Autoantibody discoveries offer a new generation of potential biomarkers to characterize a wider range of post-translational modifications caused by stress on cells and tissues prior to the development of a diagnosed autoimmune pathology. These methods allow direct observations from human specimens that can uncover unique fragments with biological responses. The discovery of the adiponectin receptor binding to IDE results was directly from the proteomic analysis of the AdipoR1 CTF in plasma [14]. Circulating autoantibodies recognizing this fragment (IgG-CTF) appear in most human and animal blood and were measurable by ELISA in all patients included in this study [18]. No significant differences in IgG-CTF values in the diabetes or CVAD disease groups were found compared to healthy controls, which is in agreement with earlier results [18].

The presences or absence of IgG-CTF autoantibodies also did not correlate significantly with accelerated progression to T2D and CV but correlated to lower risk of mortality. These findings suggest that IgG-CTF autoantibodies have general neutralizing function, potentially eliminating the ability of CTF peptides to inhibit IDE in the blood. Verification of this new autoantigen in human blood is difficult to translate because the CTF binds other molecules and is only transiently present at very low concentrations, making reproducible measurements difficult to achieve in patient samples [18]. However, the measurement of autoantibodies recognizing this proteolytic fragment could potentially indicate receptor turnover due to disease stress early in a patient’s clinical course. Patients on therapies inhibiting release of Tumor Necrosis Factor TNFα have been shown to have suppressed IgG-CTF autoantibodies while some cancer and liver disease patients have elevated values [18].

Autoantibody responses can be measured in patients over longer periods of time without seeing significant change. Previous studies showed no variation in the levels of IgG-CTF over 90 days in samples from patients without significant weight change [18]. Further work is necessary to understand the development and loss of these autoantibodies during disease. In this study, age, sex, and race did not significantly affect IgG-CTF values in patients and it also agrees with previous findings that autoantibodies in diabetes and control subjects did not correlate with diabetes progression as measured by impaired fasting glucose or impaired glucose tolerance [18]. Although IgG-CTF values do not change following glucose administration (GGTT), the free CTF autoantigen does change during the subsequent 120 min in animal models suggesting a biological role [18].

Hypertension was the expected other predictor of progression in this population and the EHR data confirmed this significant factor. Additionally, elevated levels of Bikunin and Uristatin due to inflammatory white blood cell elastase were very common in these patients (22%) and did predict increased risk of the development of comorbidities (P>0.05) in specific subsets of patients. Bikunin and Uristatin immunoassay values are typically within the normal range for the diabetic and cardiovascular population in the absence of infection, inflammation, tissue injury or kidney disease (38–40). The lack of correlation between increased white blood cell elastase and IgG-CTF values was not surprising as autoantibodies could result from multiple tissue locations, proteolytic events and normal receptor turn over. Additional work on the causes of IgG-CTF values outside of the reference range is needed to identify the value this marker.

This study confirms that retrospective analysis of biorepositories using EHR-based outcomes can provide insight into the role of autoantibodies to proteolytic fragments of cell receptors. Autoantibodies immunoassays provide stable measurements and avoid the problems of measuring transient autoantigens. A highly-multiplexed panel of antibodies covering a wide range of receptor fragments could potentially detect higher receptor turnover in tissues as a more comprehensive multi-factorial receptor response during disease. Genetic blood assays often cannot resolve signals from the post-translational fragmentation of receptors. Here, autoimmune assays may help to resolve phenotypic expression profiles in tissues that cannot be investigated by blood gene testing.

## Figures and Tables

**Table 1:** Patient demographics from electronic medical records at and after sample collection.

		Cohorts or Affect Groups^a^			

	Total	Control	T2D (no CV)	CV (no T2D)	CV+T2D

**N**	**1862**	**733**	**427**	**344**	**358**

**N**, Sample + 5yrs		607	415	340	500

Gender, M (%)	943 (50.6%)	278 (37.9%)	210 (49.2%)	245 (71.2%)	210 (58.7%)
Gender, F (%)	919 (49.4%)	455 (62.1%)	217 (50.8%)	99 (28.8%)	148 (41.3%)
Age (SD)	56.1 (9.7)	54.6 (9.3)	54.2 (9.3)	58.5 (9.8)	59.3 (9.8)
Age Range	[22 – 83]	[34 – 82]	[36 – 82]	[30 – 83]	[22 – 82]
Race, White (%)	1525 (81.9%)	551 (75.2%)	350 (82.0%)	305 (88.7%)	319 (89.1%)
Race, Black (%)	100 (5.4%)	35 (4.8%)	23 (5.4%)	15 (4.4%)	27 (7.5%)
Race, Other/Unknown (%)	237 (12.7%)	147 (20.1%)	54 (12.6%)	24 (7.0%)	12 (3.4%)
# Deaths (%)	68 (3.6%)	11 (1.5%)	8 (1.6%)	15 (4.4%)	34 (9.5%)
Age at Death (SD)	67.4 (12.7)	68.7 (13.3)	74.9 (12.2)	70.1 (11.1)	64.2 (12.7)

**Medications (Filled)** ^b^					
Anti-inflammatory	751 (40.3%)	251 (34.2%)	184 (43.1%)	142 (41.3%)	174 (48.6%)
Anti-hypertensive	705 (37.9%)	64 (8.7%)	243 (56.9%)	184 (53.5%)	214 (59.8%)
Hyperlididemic	510 (27.4%)	32 (4.4%)	176 (41.2%)	142 (41.3%)	160 (44.7%)
Anti-thrombotic	62 (3.3%)	12 (1.6%)	8 (1.9%)	13 (3.8%)	29 (8.1%)
Glucose lowering	461 (24.8%)	0 (0.0%)	275 (64.4%)	0 (0.0%)	186 (52.0%)

**Diagnoses** ^c^					
Type 2 Diabetes	644 (34.6%)	0 (0.0%)	301 (70.5%)	0 (0.0%)	343 (95.8%)
Cardiovascular	702 (37.7%)	0 (0.0%)	0 (0.0%)	344 (100.0%)	358 (100.0%)
Chronic Kidney Disease	106 (5.7%)	3 (0.4%)	12 (2.8%)	20 (5.8%)	71 (19.8%)
Liver Disease	60 (3.2%)	4 (0.5%)	21 (4.9%)	7 (2.0%)	28 (7.8%)
Obese	315 (16.9%)	30 (4.1%)	79 (18.5%)	46 (13.4%)	160 (44.7%)
Hyperlididemia	972 (52.2%)	88 (12.0%)	239 (56.0%)	305 (88.7%)	340 (95.0%)
Hypertension	786 (42.2%)	40 (5.5%)	184 (43.1%)	244 (70.9%)	318 (88.8%)

**Diagnoses** ^c^, Sample + 5yr**s**					
Type 2 Diabetes	798 (42.9%)	0 (0.0%)	323 (77.8%)	0 (0.0%)	475 (95.0%)
Cardiovascular	840 (45.1%)	0 (0.0%)	0 (0.0%)	340 (100.0%)	500 (100.0%)
Chronic Kidney Disease	207 (11.1%)	3 (0.5%)	20 (4.8%)	29 (8.5%)	155 (31.0%)
Liver Disease	106 (5.7%)	6 (1.0%)	33 (8.0%)	14 (4.1%)	53 (10.6%)
Obese	462 (24.8%)	38 (6.3%)	119 (28.7%)	48 (14.1%)	257 (51.4%)
Hyperlididemia	1176 (63.2%)	120 (19.8%)	275 (66.3%)	300 (88.2%)	481 (96.2%)
Hypertension	1007 (54.1%)	51 (8.4%)	229 (55.2%)	266 (78.2%)	461 (92.2%)

a Comparisons were made for control (unaffected) population and the affected group with type 2 diabetes (T2D), cardiovascular disease (CV) or both (CV+T2D). The number of patients with the parameter and the % of population with the parameter are shown

b Diabetic medication included glucose lowering and insulin. Anti-inflammatories include aspirin (ASA) and non-steroidal anti-inflammatories (NSAIDs). Lipid-reducing medications include statins. Anti-hypertensives include ACE inhibitors and/or beta blockers

c Clinical diagnosis based on ICD9 and ICD10 codes were used for T2D, CV, Chronic Kidney Disease (CKD), & Liver disease (LD). A systolic BP > 140 mmHg, a diastolic BP > 90 mmHg, and/or the use of anti-hypertensives defines a hypertensive patient. Patients with a value >5 and/or using lipid reducers are defined as hyperlipidemic. Obesity defined as a body mass index (BMI) > 30. The number and % for T2D, CV and CV+ T2D clinical diagnosis are shown at time of sample collection and 5 years from after sample collection.

**Table 2:** Diagnostic testing obtained from electronic medical records during the patient care.

		Control Group	T2D (no CV)	CV (no T2D)	CV+T2D
	
	N	733	427	344	358
	
Parameter^a^	Units				

HbA1c	%	6.03 (0.55) n = 50	7.53(1.54) n=306***	5.80(0.37) n = 73**	7.40(1.67) n =271***
Fasting Glucose	mg/dL	80.0 (6.97) n = 4	159.5 (65.4) n = 6	97.3 (24.3) n=5	185.0 (158.0) n=5
Glucose	mg/dL	100.1 (21.2) n = 99	172.4(69.8) n=80***	108.6 (16.8) n=170***	156.3(59.3) n=228***
Diastolic Blood Pressure	mmHg	73.0 (11.1) n = 37	74.8 (9.3) n = 30*	72.4 (8.5) n = 74	71.0 (11.5) n=109
Systolic Blood Pressure	mmHg	123.2 (17.3) n = 37	135.3 (13.5) n = 30	124.5 (14.9) n = 74	129.2 (17.3) n=109
Alanine Transaminase (alt)	U/L	28.7 (28.5) n = 58	29.8 (18.18) n = 56	29.1 (14.4) n = 133	27.8 (14.9) n =160
Aspartate Transaminase (ast)	U/L	30.5 (29.5) n = 54	30.4 (15.6) n = 53	32.9 (24.8) n = 114	35.7 (47.4) n =144
Direct Bilirubin	mg/dL	0.13 (0.076) n = 7	0.122 (0.1) n = 10	0.114 (0.118) n=39	0.112 (0.072) n=7
eGFR	mL/min/1.73m^2^	66.0 (17.2) n = 87	64.8 (16.5) n = 72	60.7 (9.82) n=159**	56.4 (14.8) n =211***
Total Billirubin	mg/dL	0.65 (0.27) n = 51	0.56 (0.31) n = 49	0.60 (0.34) n =87	0.56 (0.34) n = 128
Albumin	g/dL	4.13 (0.43) n = 53	3.91 (0.57) n = 51	4.01 (0.49) n=88	3.71 (0.52) n=143*
Creatinine	mg/dL	0.96 (0.44) n = 94	0.96 (0.48) n = 73	0.99 (0.26) n=171	1.17 (0.74) n=221**
Albumin Creatinine Ratio	mg/g	None	196.4 (149.9) n = 6	None	391.6 (990.2) n = 23
BUN	mg/dL	15.1 (5.7) n = 90	16.4 (8.0) n=69	15.8 (5.6) n =164	20.4 (11.2) n = 218***
Total Cholesterol	mg/dL	182.6 (34.4) n = 54	162.1 (45.0) n=29*	167.0 (39.8) n =161*	164.7 (45.3) n = 151*
HDL cholesterol	mg/dL	54.4 (18.5) n = 51	37.7 (11.9) n =27***	42.6 (12.8) n =159***	39.5 (11.2) n = 150***
LDL cholesterol	mg/dL	100.5 (26.0) n = 235	90.4 (31.7) n =282***	93.1 (34.2) n =243***	87.1 (33.8) n =277***
Triglycerides	mg/dL	117.5 (67.0) n=47	181.3 (110.7) n=27**	157.7 (112.8) n=155*	181.2 (114.9) n=147**
Basophil Count	k/mcL	0.029 (0.040) n = 52	0.043 (0.058) n= 38	0.036(0.047) n=99	0.039(0.047) n=130
Neurophil Count	k/mcL	4.19 (1.98) n = 54	5.64 (3.55) n=41*	5.91 (2.57) n=106***	5.86 (2.25) n=128***
Eosinophil Count	k/mcL	0.164 (0.117) n = 53	0.176(0.127) n=39	0.224 (0.210) n=101	0.188(0.125) n=130
Lymphocyte Count	k/mcL	1.84 (0.60) n=54	2.06 (0.89) n=3	1.96 (0.90) n=105	1.94 (0.75) n=130
Monocyte Count	k/mcL	134.8 (54.8) n = 577	100.1 (21.2) n=99**	172.4(69.8) n=80*	108.6 (16.8) n=170***
Platelet Count	k/mcL	239.4 (65.4) n = 86	263.6 (77.2) n = 61*	237.1 (70.6) n = 160	249.5 (76.5) n = 200
Protein	g/dL	7.08 (0.60) n = 52	7.21 (0.51) n = 49	6.97 (0.62) n = 88	6.88 (0.73) n = 130
Troponin	ng/ml	0.013 (0.021) n = 10	0.016 (0.019) n = 7	0.098 (0.200) n = 18	1.17 (6.51) n = 39

a Comparisons of mean, standard deviation and counts, were made for control (unaffected) population and the affected group (T2D), cardiovascular disease (CV) or both (CV+T2D) are shown for diagnostic measurements record in the 2019 electronic health record after the initial sample collection was obtained.

* p values of 95% significance for difference observed between the proportion in the affected and in the control groups.

** p values of 99% significance.

*** p values of 99.9% significance.

**Table 3:** Comparison of Ig-CTF and uristatin reference ranges for values from biorepository samples.

**A.** Autoimmune antibody to C-terminal fragment of adiponectin receptor (IgG-CTF) values in plasma samples *												

	Total	IgG-CTF values ≤ 821 ng/mL*					IgG-CTF values > 821 ng/mL*					

Patient group ^†^	n	Mean	SD	min	max	%	Mean	SD	min	max	n	%
All	1778	242.4	153.4	0.0	820.5	97.5%	1655.8	1415.2	829.1	7892.0	45	2.5%
Control Group	684	261.9	155.4	10.7	811.0	38.5%	1395.0	818.9	829.1	3710.8	16	0.9%
CV (no T2D)	341	243.0	164.0	0.8	820.5	18.6%	1372.3	460.0	905.2	2387.7	11	0.6%
T2D (no CV)	403	220.8	141.5	0.0	778.9	22.2%	1475.1	1119.5	904.1	4236.4	8	0.4%
CV+T2D	350	228.7	146.8	0.3	801.2	19.7%	2529.7	2523.3	832.6	7892.0	10	0.6%

	Total	IgG-CTF values > 75 ng/mL*					IgG-CTF values 75 ng/mL*					

Patient group ^†^	n	Mean	SD	min	max	%	Mean	SD	min	max	n	%
All	1778	306.7	359.3	75.4	7892.0	89.1%	43.7	19.9	0.0	75.0	193	10.8%
Control Group	684	308.1	261.0	75.4	3710.8	35.5%	48.8	17.5	10.7	74.9	52	2.9%
CV (no T2D)	341	317.6	272.0	78.1	2387.7	16.5%	46.4	20.1	0.8	73.5	48	2.7%
T2D (no CV)	403	273.8	274.2	76.4	4236.4	17.2%	37.5	21.5	0.0	73.9	48	2.7%
CV+T2D	350	331.8	608.9	76.5	7892.0	19.7%	41.4	19.0	0.3	75.0	45	2.5%

**B.** Anti-inflammatory Uristatin values in urine sample ^‡^												

	Total	Uristatin values ≤ 7.5 mg/L^†^					Uristatin values > 7.5 mg/L^†^					

Patient group ^†^	n	Mean	SD	min	max	%	Mean	SD	min	max	n	%
All	790	2.1	1.8	1.0	7.3	83.9%	16.2	25.9	7.6	278.9	127	16.0%
Control Group	321	2.2	1.8	1.0	7.0	35.9%	11.5	5.1	7.6	37.2	37	4.7%
CV (no T2D)	7	3.1	2.7	1.0	7.0	0.9%	NA	NA	NA	NA	NA	NA
T2D (no CV)	379	2.1	1.8	1.0	7.3	48.0%	18.4	33.4	7.7	278.9	73	9.2%
CV+T2D	83	2.3	2.0	1.0	7.2	8.3%	16.5	12.1	8.1	53.3	17	2.2%

**C.** Anti-inflammatory Bikunin values in plasma samples												

	Total	Bikunin values ≤ 1.4 μg/mL					Bikunin values > 1.4 μg/mL					

Patient group ^†^	n	Mean	SD	min	max	%	Mean	SD	min	max	n	%
All	1713	0.63	0.31	0.00	1.40	93.1%	1.71	0.26	1.40	2.59	119	6.9%
Control	655	0.71	0.32	0.05	1.40	34.3%	1.73	0.29	1.41	2.60	67	3.9%
CV (no T2D)	325	0.65	0.28	0.09	1.38	17.7%	1.60	0.14	1.42	1.90	21	1.2%
T2D (no CV)	392	0.56	0.21	0.00	1.36	21.9%	1.72	0.25	1.40	2.25	16	0.9%
CV+T2D	350	0.56	0.31	0.00	1.39	19.0%	1.75	0.29	1.43	2.43	15	0.9%

* IgG-CTF values were measured by sandwich immunoassay using antibodies specific for CTF and human IgG directly conjugated to alkaline phosphatase [18]. The 821 ng/mL threshold for abnormally high levels was based on 273 ng/mL mean + three standard deviations (183 ng/mL) of all samples except 2.5% over 1300 ng/mL. Samples below 75 ng/mL were considered abnormally low levels and were ∼11% of all samples.

† All values are from the time of initial sampling (2007–2011) and patients were assigned to the control, CV (no T2D), T2D (no CV) and CV+T2D groups. Urine samples were not collected from most CV patients.

‡ Uristatin and bikuinn values were measured by immunoassay using antibodies specific for uristatin and bikunin [37]. The threshold for abnormal levels were previously set based on 98% reference range established from adults and patients [38].

**Table 4:** Prediction of additional co-morbidities.

**A.** Odds ratios for patient progression to any additional co-morbidities for patients with high blood pressure, hyperlipidema and biomarkers outside of reference ranges. Most are not statistically significant due to small number of observations, but for those that are statistically significant are shown by the number of asterisks.							

		Uristatin ≥7.5 mg/L	Bikunin ≥1.4 mg/L	HT	HLD	IgG-CTF ≤75 ng/mL	IgG CTF ≥821 ng/mL

Control Group	Odds ratio	1.42	0.58	2.78**	1.07	1.26	1.20
	n (all tested)	320	653	722	721	686	686
	n (all progressing) ^†^	40	105	117	117	111	111

CV (no T2D)	Odds ratio	NS	1.2	2.06**	0.56	1.05	0.88
	n (all tested)	7	326	344	344	343	343
	n (all progressing) ^†^	2	68	69	69	69	69

T2D (no CV)	Odds ratio	1.23	1.95	0.75	0.39***	1.48	1.93
	n (all tested)	380	391	425	425	403	403
	n (all progressing) ^†^	116	135	144	144	139	139

CV+T2D	Odds ratio	0.54	0.53	1.77	2.47	0.73	0.87
	n (all tested)	83	341	357	357	351	351
	n (all progressing) ^†^	15	75	78	78	78	78

Death	Odds ratio	1.04	1.50	5.54*	2.78***	1.87	0.0
	n (all tested)	792	1713	1849	1849	1785	1785
	n (all progressing) ^†^	18	60	67	67	61	61

**B.** Additional significant (p value < 0.05) odds ratios for transitions from initial cohort condition to additional states					

Biomarker Condition	Cohort	Post-Sample State	Odds Ratio	n (all tested)	n (all progressing)

Hypertension - HT	Control Group	CV (no T2D)	2.58*	722	70
Hypertension - HT	Control Group	Liver Disease	9.39**	722	9
Hypertension - HT	T2D (no CV)	Death	8.25*	425	144
Uristatin≥7.5 mg/L	T2D (no CV)	CV+Liver Disease	13.11*	425	5
Hyperlipidemia - HLD	CV (no T2D)	Chronic Kidney Disease	0.37*	344	24
Bikunin ≥1.4 mg/L	CV (no T2D)	Death	4.45*	344	15

† Patients who developed diabetes, cardiovascular disease (CVAD), chronic kidney disease (CKD) or liver disease (LD) any time after sampling were considered as the combined group of patients progressing to comorbidities. Patient deaths in all four patient groups were combined to allow significance testing. NS = not suitable for significance testing due to sample size (CVAD urine samples, only n=7).

* p values of 95% significance.

** p values of 99% significance.

*** p values of 99.9% significance
